# Health Conditions and Risk Factors in TROVAILMIOVACCINO Users: A Study Promoting Adult Vaccination

**DOI:** 10.3390/vaccines13101025

**Published:** 2025-09-30

**Authors:** Cristina Salvati, Marco Del Riccio, Marcello Settembrini, Alessio Radi, Paolo Bonanni, Sara Boccalini, Angela Bechini

**Affiliations:** 1 Department of Health Sciences, University of Florence, 50134 Florence, Italy; marco.delriccio@unifi.it (M.D.R.); paolo.bonanni@unifi.it (P.B.); sara.boccalini@unifi.it (S.B.); angela.bechini@unifi.it (A.B.); 2 Medical Specialisation School of Hygiene and Preventive Medicine, University of Florence, 50134 Florence, Italy; marcello.settembrini@unifi.it (M.S.); alessio.radi@unifi.it (A.R.)

**Keywords:** vaccine, immunization, digital health, adults, vulnerable population, healthcare providers

## Abstract

**Background/Objectives**: The “TROVAILMIOVACCINO” platform was developed to help adults in Italy identify vaccines recommended for them based on individual characteristics, in line with the Italian National Immunization Plan (NIP). The website directs users to an anonymous online questionnaire addressing key factors such as age, sex, pregnancy status, travel history, medical conditions, and risky behaviors. It is intended for adults aged 18 and over and can be filled out either by individuals or by others on their behalf, such as healthcare professionals. The purpose of the study was to assess the platform’s reach, the health status of users, and its ability to inform users. **Methods**: Data were organized into tables and analyzed using frequencies, percentages, and statistical tests to assess user demographics and health conditions. Significant differences among sociodemographic groups were evaluated using the Chi-square and Fisher’s exact tests. **Results**: Over 30 months, the website was accessed 1897 times, with 1622 users (85.5%) completing the questionnaire for personal interest. The majority of users were aged 18–49 years (61.5%), with a nearly equal male–female distribution. Healthcare workers represented the most common professional group (29.2%) among users. Older individuals were more likely to have the questionnaire completed by someone else. Among respondents, 25.8% reported having a single medical condition, with cardiovascular diseases (11.9%), diabetes (6.7%), and respiratory diseases (4.8%) being the most frequent. The most common risk condition reported was potential contact with newborns. **Conclusions**: The findings highlight the value of the platform in reaching diverse user groups and offering tailored vaccine recommendations.

## 1. Introduction

Italy has a well-established history of successful vaccination programs, but there is still room for improvement. Starting in 2013, a decline in vaccination rates was observed, particularly affecting childhood immunization coverage. This prompted the Italian government to introduce compulsory pediatric vaccinations in 2017 through the National Immunization Plan (NIP) 2017–2019 and Law 119/2017 [1,2,3,4,5]. While these measures successfully addressed the gap in pediatric coverage [6], adult vaccination rates have remained low and are often poorly monitored [7]. Achieving high vaccination compliance is essential to ensure the health and well-being of the population, especially of at-risk adults, and to prevent the resurgence of vaccine-preventable diseases [8].

Vaccine hesitancy, driven by a combination of factors including a lack of confidence, misinformation, and sociocultural influences, poses a significant challenge to vaccination efforts [9,10,11,12]. Although it is widely recognized that vaccine hesitancy is not only attributable to a lack of information (the information deficit model), insufficient knowledge about recommended vaccines remains a critical issue among both the general population and healthcare providers (HCPs) [13,14]. This gap in understanding can further fuel doubts and concerns, underscoring the need for accessible, accurate, and personalized vaccine information. HCPs remain a critical point of reference for addressing vaccine-related concerns and should always play a central role in patient education. However, integrating a range of solutions alongside traditional care, including digital tools, is essential to maximize outreach and ensure that accurate and personalized vaccination information is available to all. Digital platforms offer several advantages, including improved access to health information, real-time updates, and customized guidance based on user-specific data. In the context of vaccination, these tools have the potential to bridge gaps in communication between HCPs and the public, address misinformation, and integrate the process of identifying individual vaccine needs. TROVAILMIOVACCINO (www.trovailmiovaccino.it (accessed on 10 June 2025) [15] is a digital platform created to facilitate the identification of recommended vaccines for adults from the Italian NIP based on personal characteristics. The website is designed for both citizens and HCPs, offering tailored vaccine recommendations based on age, gender, medical conditions, occupation, and specific lifestyles.

This study aims to profile individuals using the TROVAILMIOVACCINO website from 2021 to 2024 by analyzing user demographics, health conditions, and professional backgrounds, while identifying peak usage periods and comparing patterns between HCPs and the general public. The goal is to understand the platform’s outreach and its potential effectiveness in disseminating vaccine information.

## 2. Materials and Methods

### 2.1. The Questionnaire: Design and Validation

The website leads to an anonymous questionnaire (Supplementary Material S1) consisting of seven questions. This survey was designed and identified as a minimum number of questions through which it is possible to locate the recommended vaccines, based on the 2017–2019 NIP [5]. The approach is similar to that of the Adult Vaccine Assessment Tool promoted by the Centers for Disease Control and Prevention CDC [16].

The survey is designed for adult people. The questions cover significant demographic and health-related factors. Each section of the questionnaire was designed to inform risk stratification (by pathology, by occupational exposure, by behavior, etc.) for vaccine-preventable diseases. The first question assessed whether the respondent was completing the questionnaire for themselves or on behalf of someone else, such as a patient (in the case of healthcare providers). Age was categorized into three groups (18–49, 50–64, and over 65 years), while sex was recorded as either male or female. Based on the selected sex, additional context-specific questions were generated: females were asked to indicate pregnancy status (pregnant, postpartum, or neither), whereas males were asked whether they have sex with other men (MSM) due to its relevance in identifying specific health risk factors.

Participants were then asked to indicate the presence of any underlying medical conditions that could influence susceptibility to infectious diseases. These included cardiovascular diseases, type 1 or type 2 diabetes, chronic respiratory diseases (including asthma requiring high-dose oral corticosteroids), renal failure or dialysis, chronic liver disease, absence or dysfunction of the spleen, complement deficiency, current or planned immunosuppressive therapy, active cancer treatment (chemotherapy or radiotherapy), history of bone marrow or solid organ transplantation, HIV infection, AIDS, cochlear implants, immunoglobulin or cellular immunodeficiencies, and a history of malignant neoplasms. Respondents were also asked if they cohabited with individuals affected by these conditions.

Travel history was considered by inquiring whether the participant had any plans to travel abroad, an important determinant of vaccine-preventable disease exposure. Occupational risk was assessed through a comprehensive list of professions known to be associated with increased exposure to biological agents. These included healthcare workers, school employees, professionals working with animals or animal products (e.g., farmers, veterinarians), personnel in critical public service roles (e.g., police forces, firefighters), individuals handling waste or involved in funeral services, tattoo artists and body piercers, and laboratory workers handling biological materials.

Finally, the questionnaire included questions targeting specific behavioral or social risk factors. These included intravenous drug use, cohabitation with hepatitis B-positive individuals, sexual intercourse with sex workers, incarceration, chronic alcohol use, blood donation, and expected contact with neonates.

For some questions, it was possible to select more than one option such as in the “professional category” or “conditions” sections. Moreover, for all questions, an answer is required, and therefore at least one option (including “none of the above” in the case of risk conditions and comorbidities) must be indicated, otherwise it is not possible to complete the survey. Upon completing the questionnaire, it is possible to download and save a PDF document containing all the recommended vaccines for the selected personal characteristics. The final summary page also contains an explanation of why each suggested vaccine is recommended specifically for the subject who has selected certain conditions and the scientific rationale. The summary document of recommended vaccinations can be sent to one’s family doctor for clarification requests, for the verification of their vaccination status, and for planning any booster shots, facilitating a constructive dialog between the doctor and the patient. No indication is given as to where to receive the vaccines. In general, vaccines are available and administered at General Practitioners’ (GPs) clinics, local health authorities, and travel medicine clinics, but the territorial organization of vaccination services also changes according to the different Italian regions.

After designing the questionnaire and developing a beta version of the website, a validation process was conducted to ensure the tool’s accuracy and functionality. This process involved 10 medical residents from the Public Health School at the University of Florence who verified that all recommendations provided by the tool were consistent with the 2017–2019 National Immunization Plan (NIP) and that no errors or inconsistencies were present in the vaccine identification process. In addition, technical testing was carried out to ensure that the website functioned correctly across different platforms and devices, guaranteeing that users could easily access the tool without encountering technical malfunctions. Feedback from early testers was incorporated to refine both the content and user experience before the official launch. TROVAILMIOVACCINO was mainly disseminated in the network of Hygiene and Public Health experts in Italy, on the institutional channel of the Italian Society of Hygiene, and to GPs. In addition, the tool was presented on the online portals of https://www.Vaccinarsi.org (both regional and national) and through posters and abstracts at conferences dedicated to HCPs working in the field of vaccine-preventable diseases.

### 2.2. Inclusion and Exclusion Criteria

The study’s inclusion criteria include all those people aged 18 years or over who filled out the questionnaire, as people under that age are not allowed to fill in the questionnaire. Age ranges of the questionnaire were established in accordance with the recommendations of the NIP. Even if the questionnaire was completed by an HCPs or another person on behalf of someone else, the patient must be an adult, as the questionnaire is intended exclusively for the adult population. This is because, in Italy, the age of 18 is the legally recognized threshold for adulthood. Conventionally, adolescents up to the age of 13 are cared for by a pediatrician, while from the age of 14 they are assigned to a GP. This represents an important transition age, during which vaccination recommendations also change accordingly.

### 2.3. Statistical Analysis

All questionnaires completed anonymously between July 2021 and January 2024 were exported. Data were collected in a dedicated spreadsheet and subsequently organized into tables for analysis. Frequencies and percentages were calculated for all categories, including user demographics (e.g., filling in for oneself or on behalf of others, sex, and profession). Additionally, the number of users who reported at least one risk condition or more than one pathology was determined. The analysis also included identifying how many users had specific health conditions (e.g., cardiovascular disease, diabetes). To assess any significant differences among the considered sociodemographic groups, the Chi-square test and Fisher’s exact test for the comparison between dichotomous variables were applied. A significance level of *p* < 0.05 was considered statistically significant. All analyses were conducted using Rstudio version 2023.06.0+421 (Posit Software, PBC, Boston, MA, USA, http://www.posit.co/ (accessed on 10 June 2025).

## 3. Results

The questionnaire was completed by 1897 individuals between July 2021 and January 2024. Of these, 51.5% (977 users) were male and 48.5% (920 users) were female. Regarding the age distribution, the majority of respondents fell into the 18–49 years age group, accounting for 61.5% (1166 users) of the total. The 50–64 years age group represented 24.7% (468 users), while the over 65 years age group comprised 13.9% (263 users). The majority of users filled out the questionnaire for themselves (1622 users, 85.5%), while 14.5% (275 users) completed it on behalf of others. Younger users were more likely to fill out the questionnaire for themselves, while older users more frequently had the form filled out on their behalf (*p* < 0.001 with Chi-square test applied). In the 18–49 years age group, 90.7% of users completed the form for themselves, compared to only 9.3% who had it completed by someone else. Similarly, in the 50–64 years age group, 82.9% filled it out for themselves and 17.1% did so for others (Table 1).

Among the 920 women who completed the questionnaire, 35 (3.8%) reported being currently pregnant, while 18 (2.0%) were in the puerperium period. The remaining 862 women (93.7%) were not pregnant. Among the 977 male users, 234 (24.0%) reported having sexual intercourse with other men. Excluding those who reported not belonging to any specific occupation that might require targeted vaccination, the two most frequently reported occupations were healthcare workers (525 users, 29.2%) and school workers (114 users, 6.3%). Regarding traveling abroad, 843 (44.4% out of the total) expressed interest, indicating a substantial proportion of individuals considering international travel that may require specific vaccination.

**Figure 1:**
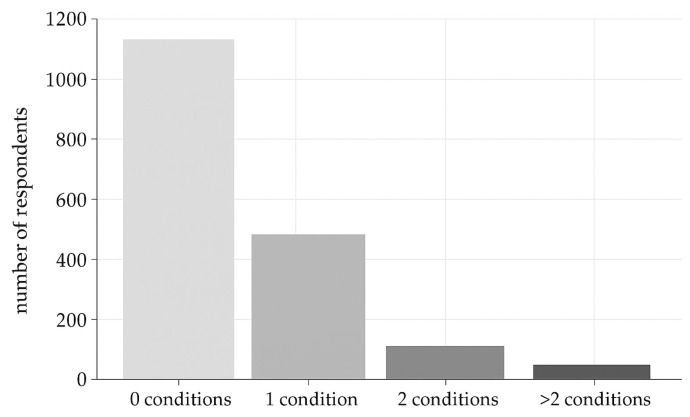
Distribution of respondents based on the number of conditions reported (0, 1, 2, or more than 2 conditions).

About health conditions and pathologies, 490 respondents (25.8%) reported only one condition, 102 (5.4%) reported two, and 70 (3.7%) reported more than two (Figure 1).

**Figure 2:**
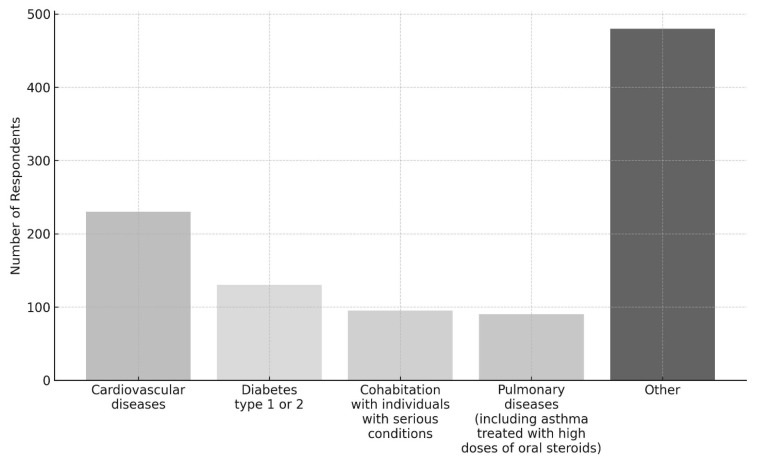
Distribution of the top four reported conditions and other conditions among respondents.

In particular, the most commonly reported conditions were cardiovascular diseases (227 users, 11.9%), diabetes type 1 or 2 (128 users, 6.7%), and pulmonary diseases (91 users, 4.8%). Moreover, 96 users (5.0%) reported cohabitation with individuals affected by serious conditions (Figure 2).

In addition, those who were currently taking immunosuppressive medications numbered 87 (4.6%), and 41 individuals (2.2%) were expected to start such therapy. Chronic liver diseases were present in 51 respondents (2.7%), kidney failure or dialysis in 46 (2.4%), HIV in 50 (2.6%), and malignant neoplasms in 44 (2.3%). Cancer patients undergoing chemotherapy or radiotherapy totaled 20 (1.1%), while 26 people (1.4%) had a damaged or removed spleen. Organ or bone marrow transplant recipients were 25 (1.3%), cochlear implant carriers were 13 (0.7%), and immune system disorders, such as dysgammaglobulinemia or cellular immunity deficits, affected 19 individuals (1%). Finally, rare conditions like complement deficiencies and AIDS each accounted for 9 cases (0.5%).

The majority of respondents (1470, 78.5%) selected “none of the above” when asked about other possible specific risk conditions that may require vaccination, while 164 respondents (8.8%) indicated that they expect to come into contact with newborns, and 122 respondents (6.5%) reported being blood donors. In addition, among the people who selected a single risk condition, 45 (2.4%) reported having sexual intercourse with sex workers, 13 (0.7%) reported drug addiction or injecting drug use, and 18 (0.9%) reported chronic alcoholism.

## 4. Discussion

The objective of this work is the evaluation of the data provided by TROVAILMIOVACCINO users, with the aim of outlining the characteristics of people seeking information on vaccines recommended at a national level in a digital tool.

In Italy, TROVAILMIOVACCINO is the first tool that allows any type of user (both citizens and HCPs) to identify recommended vaccines, thus responding to a need for accurate and precise information. A similar tool, the CDC’s Adult Vaccine Assessment Tool, is also available in the United States, and although specific studies on the use of this toolkit are not published, the literature suggests that self-assessment tools may be useful in identifying necessary vaccinations [16]. However, it is relevant to complement these tools with medical consultations and medical record reviews to ensure a thorough and individualized assessment of vaccination needs [17]. Though TROVAILMIOVACCINO is not intended to replace clinical judgment or the review of individual medical and vaccination history by a health professional, it serves as a pre-assessment and educational tool that enhances user awareness, supports informed preparation for medical consultations, and helps bridge the information gap between citizens and HCPs but also between the citizens and institutional recommendations for adult vaccination by the Ministry of Health, ultimately facilitating immunization goals.

The analysis of TROVAILMIOVACCINO users’ data (1897 individuals in 30 months) made it possible to identify, among the different categories of adults for whom vaccinations are foreseen, a significant utilization of the tool by HCPs (29.2%). Notably, HCPs emerged as a particularly active user group, not only consulting the tool to assess their own vaccination needs but also using it on behalf of others. This underscores the role of digital tools in assisting HCPs with vaccination awareness and decision-making within the healthcare sector, complementing the education of HCPs by providing up-to-date, evidence-based information on recommended vaccinations [18]. In fact, such tools can enhance public awareness of vaccine availability and be particularly valuable for HCPs in identifying recommended vaccinations for at-risk groups, ultimately contributing to increased vaccination coverage [19]. Our analysis reported a greater use of the tool by the 18–49 years age group (61.5%). Actually, several studies indicate that younger parts of the population are more inclined to search for health information online using institutional websites: this behavior may reflect a greater interest in the prevention of one’s health, as well as a greater trust in technology as a decision-making support tool [20,21,22]. Another noteworthy finding is that 44.4% of the total sample expressed interest in traveling abroad, indicating a good percentage of individuals who have considered international travel that may require specific vaccination. This data could be interpreted in the context of the post-pandemic recovery of global mobility, which has made rapid and precise access to information on travel-related health risks essential [23,24], though sources in the literature suggest that simple awareness of travel vaccines does not always result in increased vaccination compliance. Factors such as risk perception and vaccine availability may play a significant role in effective vaccination coverage among travelers [25].

In view of the above, TROVAILMIOVACCINO [15] appears to be a tool consistent with the objectives proposed by the WHO’s Immunization Agenda 2030 (IA2030) and the National Immunization Plan (NIP) 2023–2025. Both globally and nationally, there is recognition of the importance of digital tools in improving the access, management, and promotion of vaccinations [19,26]. For instance, the NIP 2023–2025 suggests the adoption of flexible digital tools and technologies to support active calling and vaccination booking management [19]. Similarly, the IA2030 places digital tools among the innovative resources that promise to transform immunization programs in the next decade, improving the management of vaccination services [26]. TROVAILMIOVACCINO could serve as a valuable tool for HCPs by supporting the identification of recommended vaccines for patients, thus acting as a further support in active calling efforts. Additionally, TROVAILMIOVACCINO could prove useful in achieving the immunization targets by providing accessible and up-to-date information on vaccines, promoting equity in access to vaccinations, and supporting the prevention of vaccine-preventable diseases.

However, this study also shows some limitations. One issue is the lack of active recruitment and large-scale promotion, which means that the sample may not be fully representative of the broader Italian population. The lack of a more diverse participant base makes it difficult to generalize the findings beyond the individuals who independently discovered and used the tool. Also, the lack of data on participants’ geographical origin (e.g., region or city of residence) restricts our ability to evaluate whether the sample is representative of the broader population. In addition, while TROVAILMIOVACCINO provides useful recommendations, it does not account for users’ vaccination history. For example, there is no question relating to the individual’s current vaccination status, which would be essential to determine whether they have already been vaccinated against certain pathogens or whether a booster is needed. Furthermore, the question about the intention to travel abroad is important, but the inclusion of a more specific question asking for the country of destination could also make it possible to provide more targeted recommendations based on the health situation of the foreign destination. Moreover, this analysis was designed as a descriptive study and does not assess behavioral or clinical outcomes resulting from use of the platform, which is primarily designed as a tool to promote vaccination; future research could build on these findings by incorporating pre–post knowledge assessments or linkage with vaccination records to determine whether the tool has a measurable impact on vaccination uptake, user knowledge, or decision-making behavior.

Moreover, to overcome the difficulty of generalizing the findings related to the lack of active recruitment and large-scale promotion, a proactive approach combining targeted digital promotion with integration into traditional healthcare pathways, in particular through collaboration with GPs, is needed. Furthermore, the inclusion of a more specific question asking for the country of destination would be a crucial improvement to provide highly customized and clinically relevant vaccine indications for international travel, which, as indicated, represents an interest for a good percentage of individuals using the platform. In addition, further attention to the matter of multiple sexual partners, regardless of gender, and the related need for vaccination would be an improvement of the questionnaire. This is because the risk of contracting sexually transmitted infections increases proportionally with the number of partners, regardless of the sexual orientation or gender of those involved. Explicitly addressing this aspect would make the population more aware of the importance of preventive vaccination and the questionnaire fairer and more comprehensive.

Despite these limitations, TROVAILMIOVACCINO is a simple and intuitive vaccination tool and can potentially reach a wide user base. The inclusion of the “fill in for others” function is particularly valuable, as it allows family members, caregivers, and HCPs to complete the questionnaire on behalf of individuals who may face digital literacy challenges, such as the elderly or people with disabilities. This feature expands the platform’s accessibility and ensures that different categories of the population have the opportunity to receive accurate vaccine recommendations. While inferential or longitudinal analyses were beyond the scope of the current design, this work provides a foundational assessment of an innovative public health tool in the digital vaccination space. As such, it might lay the groundwork for future evaluations that could assess its real-world impact on vaccine adherence or information retention. Finally, the platform successfully attracts at-risk populations, particularly younger individuals, HCPs, and travelers.

## 5. Conclusions

Targeted efforts, such as collaborations with family doctors and digital information campaigns, are needed to expand the platform’s reach to underrepresented groups, particularly those with complex health needs or with linguistic and socio-economic barriers. By applying these insights, the platform TROVAILMIOVACCINO can play a significant role in supporting national vaccination efforts and improving population-level vaccine uptake.

## Figures and Tables

**Table 1:** Distribution of users by age group who completed the questionnaire for themselves or on behalf of others.

Age Group (Years)	Self-Filled (n)	Filled by Others (n)
18–49	1058	108
50–64	388	80
≥65	176	87
Total	1622	275

## Data Availability

The dataset is available on request from the authors.

## Supplementary Materials

The following supporting information can be downloaded at: https://www.mdpi.com/article/10.3390/vaccines13101025/s1, Supplementary Material S1: Questionnaire.

## References

[1] D’Ancona F., D’Amario C., Maraglino F., Rezza G., Iannazzo S. (2019). The law on compulsory vaccination in Italy: An update 2 years after the introduction. Eurosurveillance.

[2] Di Pietro A., Visalli G., Antonuccio G.M., Facciolà A. (2019). Today’s vaccination policies in Italy: The National Plan for Vaccine Prevention 2017–2019 and the Law 119/2017 on the mandatory vaccinations. Ann. Ig..

[3] Signorelli C., Iannazzo S., Odone A. (2018). The imperative of vaccination put into practice. Lancet Infect. Dis..

[4] Ministero Della Salute Legge Vaccini. https://www.salute.gov.it/new/it/tema/vaccinazioni/legge-vaccini/.

[5] Istituto Superiore di Sanità Il Piano Nazionale di Prevenzione Vaccinale (PNPV) 2017–2019. https://www.epicentro.iss.it/vaccini/Pnpv2017-19.

[6] Rezza G. (2019). Mandatory vaccination for infants and children: The Italian experience. Pathog. Glob. Health.

[7] Epicentro Istituto Superiore di Sanità. Vaccini e Vaccinazioni.

[8] Ekezie W., Awwad S., Krauchenberg A., Karara N., Dembiński Ł., Grossman Z., del Torso S., Dornbusch H.J., Neves A., Copley S. (2022). Access to Vaccination among Disadvantaged, Isolated and Difficult-to-Reach Communities in the WHO European Region: A Systematic Review. Vaccines.

[9] Strategic Advisory Group of Experts (SAGE) Report of the SAGE Working Group on Vaccine Hesitancy. World Health Organization 2014.

[10] Geoghegan S., O’Callaghan K.P., Offit P.A. (2020). Vaccine Safety: Myths and Misinformation. Front. Microbiol..

[11] Biasio L.R. (2017). Vaccine hesitancy and health literacy. Hum. Vaccin. Immunother..

[12] Shapiro G.K., Tatar O., Dube E., Amsel R., Knauper B., Naz A., Perez S., Rosberger Z. (2018). The vaccine hesitancy scale: Psychometric properties and validation. Vaccine.

[13] Lehner L., Gribi J., Hoffmann K., Paul K.T., Kutalek R. (2021). Beyond the “information deficit model”—Understanding vaccine-hesitant attitudes of midwives in Austria: A qualitative study. BMC Public Health.

[14] European Centre for Disease Prevention and Control Vaccine Hesitancy Among Healthcare Workers and Their Patients in Europe. https://www.ecdc.europa.eu/en/publications-data/vaccine-hesitancy-among-healthcare-workers-and-their-patients-europe.

[15] TROVAILMIOVACCINO. http://www.trovailmiovaccino.it/servizi/notizie/notizie_homepage.aspx.

[16] Centers for Disease Control and Prevention The Adult Vaccine Assessment Tool. https://restoredcdc.org/www.cdc.gov/vaccines-pregnancy/resources/adult-vaccine-assessment-tool.html.

[17] Fishbein D.B., Willis B.C., Cassidy W.M., Marioneaux D., Bachino C., Waddington T., Wortley P. (2006). Determining indications for adult vaccination: Patient self-assessment, medical record, or both?. Vaccine.

[18] Lanza T.E., Paladini A., Marziali E., Gianfredi V., Blandi L., Signorelli C., Odone A., Ricciardi W., Damiani G., Cadeddu C. (2023). Training needs assessment of European frontline health care workers on vaccinology and vaccine acceptance: A systematic review. Eur. J. Public Health.

[19] Ministero Della Salute Piano Nazionale Prevenzione Vaccinale 2023–2025. https://www.trovanorme.salute.gov.it/norme/dettaglioAtto.spring?id=95963&page=newsett.

[20] Nunes C., Almeida C.V.D., Belim C. (2020). Health Literacy in Younger Age Groups: Health Care Perceptions: Informed People Will Be More Prepared People. Open Access Libr. J..

[21] Jia X., Pang Y., Liu L.S. (2021). Online Health Information Seeking Behavior: A Systematic Review. Healthcare.

[22] Minghao P., Leyun H., Jingying Y., Wanyu H., Fan W., Linlin W., Meiyu S. (2025). A grounded theory study on medical students’ proxy online health information seeking behavior. BMC Public Health.

[23] Gössling S., Scott D., Hall C.M. (2020). Pandemics, tourism and global change: A rapid assessment of COVID-19. J. Sustain. Tour..

[24] Gallego I., González-Rodríguez M.R., Font X. (2022). International air travel attitude and travel planning lead times across 45 countries in response to the COVID-19 pandemic. Tour. Manag. Perspect..

[25] Società Italiana di Medicina Generale Prevenzione Vaccinale dei Viaggiatori. https://www.simg.it/Riviste/rivista_simg/2020/01_2020/11.pdf.

[26] World Health Organization Immunization Agenda 2030: A Global Strategy to Leave No One Behind. https://www.who.int/teams/immunization-vaccines-and-biologicals/strategies/ia2030.

