# Supplementary material for: Health Conditions and Risk Factors in TROVAILMIOVACCINO Users: A Study Promoting Adult Vaccination
Source: Vaccines (Basel). 2025 Sep 30;13(10):1025. doi: 10.3390/vaccines13101025 (PMC12568262; doi:10.3390/vaccines13101025)
Supplement: Supplementary file 1 [file vaccines-13-01025-s001.zip › vaccines-3852894-supplementary.pdf]

Supplementary Material S1. Questionnaire

1. **For whom are you filling out the questionnaire?**
- For others (I am a healthcare worker)
- For myself

2. **How old are you?**
- 18-49
- 50-64
- over 65

3. **Sex:**
- Female (If the person filling in the form selects "female," the questionnaire asks whether the person is pregnant, in the puerperium, or not pregnant)
- Male (If the person filling out the form selects "male," the questionnaire asks if the person has sex intercourse with other males)

4. Select conditions:

a) Cardiovascular diseases

b) Type 1 or 2 diabetes

c) Lung diseases (including asthma if treated with high-dose oral steroids)

d) Kidney failure or dialysis

e) Chronic liver disease

f) Damaged or removed spleen

g) Complement component deficiency

h) Currently taking immunosuppressive therapy/medication

i) Planning to start immunosuppressive therapy

j) Undergoing cancer treatment with chemotherapy or radiation therapy

k) Bone marrow or solid organ transplant

l) Cochlear implant in place

m) HIV

n) Living with individuals affected by the above-mentioned conditions

o) AIDS

p) Malignant neoplasms

q) Hypogammaglobulinemia, immunoglobulin deficiency, cellular immunity deficiency

r) None of the above

5. Do you plan to travel abroad?

- Yes

- No

6. What is your occupation?

a) Healthcare worker or in the healthcare sector

b) School employee

c) Work with animals or animal-derived materials (farmers, livestock handlers, animal transporters, slaughterers, and vaccinators, public and freelance veterinarians)

d) Employee in critical public services (police forces, firefighters, military personnel)

e) Involved in waste collection, transport, and disposal

f) Tattoo artist and body piercer

g) Involved in cemetery and funeral services

h) Laboratory personnel working with biological materials

i) None of the above

7. Select if:

a) You use injectable drugs/ you are a drug user

b) You live with individuals who are Hepatitis B positive

c) You have sexual contact with sex workers

d) You are a prisoner

e) You are a chronic alcoholic

f) You are a blood donor

g) You expect to come into contact with newborns

h) None of the above
